# Cultural factors related to immigrants’ health: a scoping review

**DOI:** 10.3389/fpubh.2025.1606772

**Published:** 2025-06-18

**Authors:** Sujin Choi, Eunjeong Kang

**Affiliations:** ^1^Department of Nursing, College of Medicine, Soonchunhyang University, Asan-si, Republic of Korea; ^2^Department of Health Administration and Management, Soonchunhyang University, Asan-si, Republic of Korea

**Keywords:** immigrants, health equity, cultural factors, Donabedian model, public health

## Abstract

**Introduction:**

This scoping review investigates the cultural factors influencing the health of immigrants by applying Donabedian’s structure-process-outcome (SPO) model. While immigrant health has been extensively studied, cultural factors have not been systematically analyzed within a comprehensive healthcare quality framework.

**Methods:**

Following the Arksey and O’Malley’s scoping review framework, 42 studies were identified and analyzed using the SPO model to categorize cultural factors into structural, process, and outcome dimensions.

**Results:**

Structural factors included social support, discrimination, ethnicity, language barriers, cultural competence, and parents’ culturalism. Process factors included acculturation to new cultures, while outcome factors encompassed acculturative stress and an ethnic identity crisis. Social support and cultural competence were crucial for mitigating health challenges, whereas discrimination and language barriers were significant obstacles.

**Conclusion:**

This study highlights the importance of an integrated approach to understanding immigrant health by addressing cultural factors within a structured health model. Our findings provide actionable insights for planning culturally sensitive policies and services to enhance immigrant health outcomes.

## 1 Introduction

Immigrant populations have grown significantly worldwide, reshaping societies into multicultural landscapes. According to the Organization for Economic Cooperation and Development, a country is considered multicultural when its immigrant population, including naturalized citizens, second-generation immigrants, and foreign nationals, exceeds 5% of its total population (1). In 2024, 281 million international immigrants constituted 3.6% of the global population (2). This trend suggests that the world is moving toward establishing more multicultural societies.

In response to the emerging multicultural society, previous research has focused on education, discrimination, integration, and immigrant health. The rapid surge in immigration driven by the pursuit of improved economic opportunities, education, and public health for their families (3) has shaped perspectives on immigrants’ health issues. Although recent studies have elucidated the barriers to and facilitators of health among immigrants, they have not comprehensively identified the factors that influence health using an integrated approach. An integrated approach that acknowledges the interdependence of physical, mental, and social well-being in defining health is crucial, as stated by the World Health Organization (4). Despite this definition, research on immigrant health has traditionally focused on physical and mental health. Consequently, the holistic investigation of the factors influencing immigrants’ health remains critically underexplored.

Numerous studies have reported that immigrants face a myriad of obstacles to effective healthcare, including language barriers, legal complications, stigma, limited access to health insurance, and socioeconomic disadvantages (5, 6). However, previous studies have analyzed each influencing factor in isolation, failing to explore their interconnections within a health model (7). These factors are intricately related (8), necessitating a structural framework for their presentation. Donabedian’s model, which evaluates healthcare service quality through three components-‘structure,’ ‘process,’ and ‘outcome’ (SPO) (9), offers a robust framework to analyze the interconnected factors influencing immigrant health. By applying this model, this study sought to investigate the factors influencing physical, mental, and social well-being and provide insights into enhancing healthcare service quality.

Among the myriad factors related to immigrant health, this study focuses on cultural factors. The term culture refers to the dynamic system of shared values, beliefs, behaviors, and customs that shape how individuals perceive, interpret, and respond to health and illness (10, 11). Cultural factors shape protective behaviors and have been assessed based on immigrants’ perceptions and behaviors within specific contexts (12). Thus, an improved understanding of cultural factors is essential for developing culturally and linguistically sensitive health services that minimize health disparities among immigrants.

This study aims to systematically identify and classify cultural factors influencing the physical, mental, and social health of immigrants through a scoping review, and presents these factors using Donabedian’s SPO model (9). These findings can inform the implementation of health policies that enhance health equity in multicultural contexts.

## 2 Methods

For The methodology used in this review was based on the framework developed in 2005 by Arksey and O’Malley. A scoping review aims to clarify the research question by broadly analyzing the available materials (13). This type of review consists of five main steps and one additional optional step depending on availability (13, 14). The five main steps included identifying the research question; relevant studies; study selection; analyzing the data; and organizing, summarizing, and reporting the findings. An optional step, which is not considered in this review, involves consulting stakeholders for further insights (13). The collated, summarized, and reported factors were categorized based on Donabedian’s SPO model (9).

In this model (9), the structure included the attributes of material resources domain (such as equipment and money), human resources domain (such as the number and qualifications of personnel), and organizational resources domain (such as medical staff organization and methods of peer review and reimbursement). The process encompasses the client’s activities in seeking and receiving care as well as the provider’s efforts in making a diagnosis and recommending or implementing treatment. Outcomes included the effects of care on clients’ health status.

In this study, we define the structural category as the system or environmental factors of society, individuals, and organizations related to immigrants’ health. The process category refers to immigrants’ interactive health experiences while navigating distinct cultures. The outcome category included the consequences of immigrants’ interactive health processes. All these terms are explained along with the factors in the Results section. Ethical approval was not required due to the nature of this review.

In this study, the term “immigrant” refers to individuals who have experienced cross-national migration and resettlement, regardless of generational status. Inclusion criteria was based on whether participants had a lived experience of migration and whether cultural factors related to that migration were central to the study’s objectives. Studies focusing on long-established ethnic minority groups without migration experience were excluded.

### 2.1 Search strategies

This study provides a scoping review that explores the various cultural influences on health. The sources used in this review included ScienceDirect, CINAHL, ProQuest, PsycInfo, PubMed, Embase, Web of Science, RISS, KMbase, KoreaMed, and the Cochrane Library. Journal papers and peer-reviewed articles were searched using keywords such as (“emigrant” OR “immigrant” OR “international migrant”) AND (“health” OR “health care” OR “healthcare” OR “medical”) AND (“culture” OR “cultur” OR “acculturation” OR “ethnic*” OR “acculturation” OR “cultural belief*” OR “cultural competence”). The literature search was conducted between September 29 and October 9, 2023. During this period, iterative testing and refinement of search terms were performed to improve relevance and comprehensiveness. The final database search using the finalized keyword set was completed on October 9, 2023. The final database search was conducted by a single researcher using the finalized search strategy. Title and abstract screening, as well as full-text review, were performed independently by two researchers. Any discrepancies in study inclusion decisions were resolved through discussion and consensus.

We adopted a broad understanding of culture to capture the multifaceted influences on immigrant health. Only studies published in English or Korean in full-text were included. Exclusion criteria were: (1) studies without international migration experiences, and (2) non-original publications (e.g., editorials, letters). No restrictions were placed on study design; both qualitative and quantitative studies were included. A total of 5,236 papers were screened (based on titles and abstracts), and 85 were evaluated to determine their relevance to the research topic. Finally, 42 studies were included in the analysis.

### 2.2 Charting data

**Figure 1:**
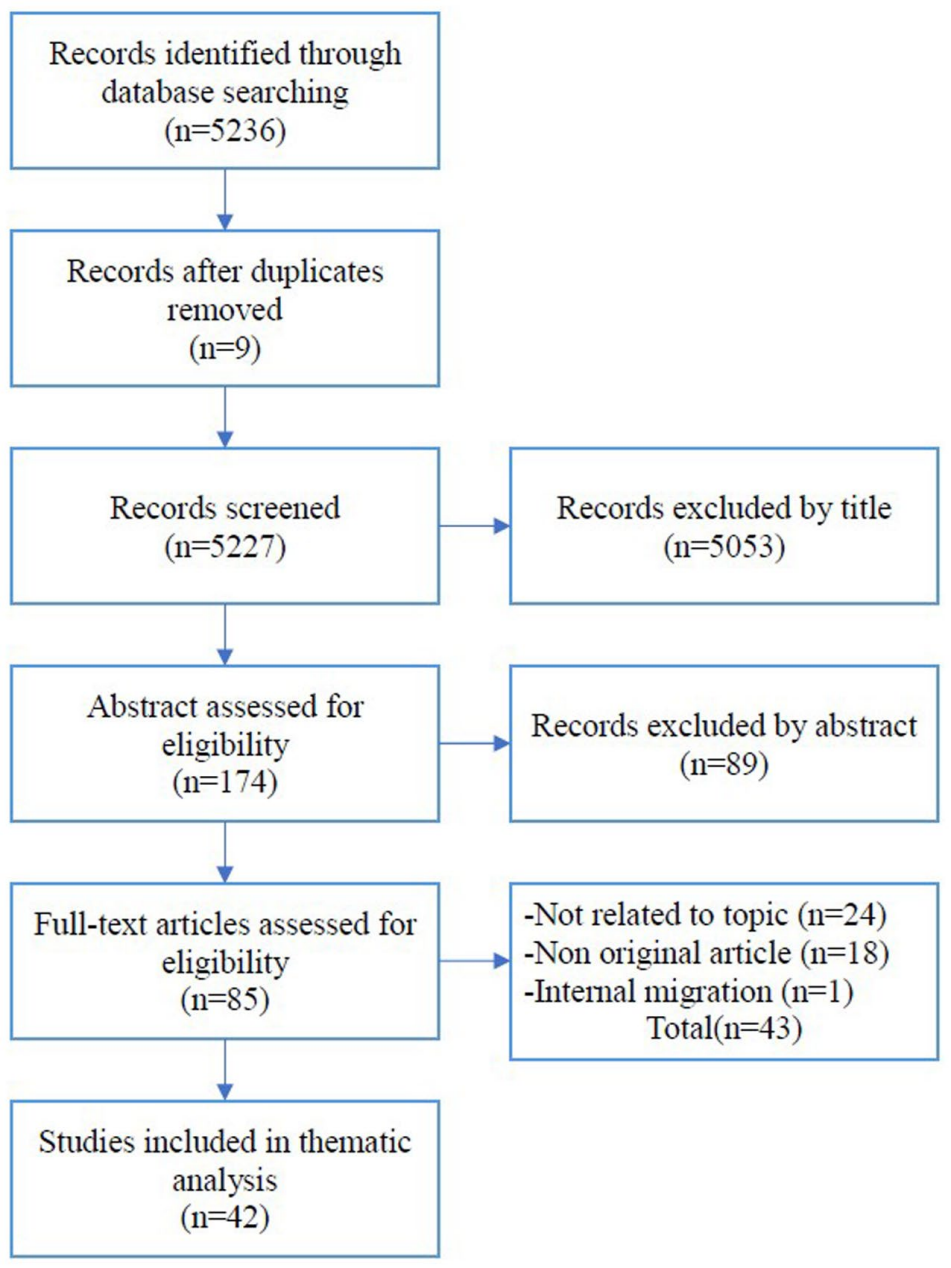
Strategy used for article selection.

After selecting the final set of studies, the pertinent data addressing the research questions were extracted. Data charting was also conducted independently by two reviewers who extracted relevant information and cross-checked results, resolving any inconsistencies by agreement. The data charting approach used is illustrated in the flowchart shown in Figure 1. The extracted data, including factors, titles, target groups, health variables, and countries, are summarized in Table 1.

**Table 1:** Analysis of selected articles.

No.	Year	Title	Factor	Target group/ Sample size	Health variable	Country
1	2012	Effects of acculturation and social network support on depression among elderly Korean immigrants	- Acculturation, - Social network support	210 Elderly Korean immigrants in Los Angeles County	Mental health	USA
2	2012	Health Beliefs and Attitudes of Latino Immigrants: Rethinking Acculturation as a Constant	- Acculturation	64 Latino immigrant adults in Miami	Physical health (reproductive health)	USA
3	2012	Immigrants and Their Health Communication Process	- Acculturation - Korean language proficiency	32 immigrants from Philippines, Sri Lanka, Vietnam, China, Mongolia, Cambodia, Uganda, Nigeria, Nepal, Bangladesh, Iran, Indonesia	Physical and mental health	South Korea
4	2014	Acculturation and Health of Korean American Adults	- Acculturation	517 Korean immigrants in a Midwestern city	Physical health and mental health	USA
5	2014	Acculturative Stress and Inflammation Among Chinese Immigrant Women	- Acculturation, - Acculturative stress	407 foreign-born Chinese American women in Philadelphia	Physical health (inflammatory markers)	USA
6	2014	Emotional acculturation predicts better somatic health: experiential and expressive acculturation among immigrant women from four ethnic groups	- Emotional acculturation	915 immigrant women from Haiti, the Dominican Republic, the English-speaking Caribbean and Eastern Europe, living in Brooklyn	Physical health	USA
7	2015	Alcohol use severity and depressive symptoms among late adolescent Hispanics: Testing associations of acculturation and enculturation in a bicultural transaction model	- Acculturation and enculturation	129 late adolescent Hispanics in the U. S.	Mental health (Alcohol use and depressive symptoms)	USA
8	2016	Acculturation and changes in body mass index, waist circumference, and waist-hip ratio among Filipino Americans with hypertension	- Acculturation	108 Filipino Americans	Physical health	USA
9	2016	Do acculturation strategies have impacts on the self-declared health, well-being and lifestyle of first-generation allophone immigrants in Montreal, Canada?	- Acculturation - Language use - Social connections - Lifestyle	506 first-generation immigrants living in the Montreal region	Physical and mental health	Canada
10	2018	Acculturation and Arab immigrant health in Colorado: a socio-ecological perspective	- Acculturation	100 adult Arab immigrants living in Colorado.	Physical health	USA
11	2019	Health risk behaviors, musculoskeletal disorders and associated cultural adaptation, depression: a survey among Myanmar migrant workers in Chiangmai, Northern Thailand	- Acculturation	414 Myanmar migrant workers in Chiangmai, Thailand	Physical and mental health	Thailand
12	2019	Pre-acculturation as a risk factor for obesity: Findings from the Health of Philippine Emigrants Study (HoPES)	- Pre-acculturation	1,632 Philippine Emigrants in the U. S.	Physical health (obesity)	USA
13	2023	Immigrant generation, acculturation, and mental health literacy among former Soviet Union immigrants in Israel	- Acculturation - Personal stigma	420 Former Soviet Union immigrants in Israel	Mental health	Israel
14	1979	The relation of blood pressure levels to the assimilation of the immigrants and intolerance of ambiguity	- Acculturative stress	50 immigrants from England, Germany, Hungary, Poland, Spain, Italy, Mexico, Nicaragua, Philippines, India, Taiwan, Egypt, Kuwait, Palestine, France, Belgium, Ireland	Physical health (cardiovascular health (highest mean blood pressure))	USA
15	2000	Acculturative Stress, Depression, and Suicidal Ideation Among Central American Immigrants	- Acculturative stress - Religion	78 American descent immigrants (El Salvador, Guatemala, Honduras, Nicaragua)	Mental health	USA
16	2006	Acculturation stress and depression among Asian immigrant elders	- Acculturation stress	407 Chinese, Filipino, Indian, Japanese, Korean, and Vietnamese immigrants	Mental health	USA
17	2011	Racial and Cultural Factors Affecting the Mental Health of Asian Americans	- Acculturative stress	367 Asian American adults (Korean, Chinese, Taiwanese, Japanese, Asian Indian, etc)	Mental health	USA
18	2008	“The Happy Migrant Effect”: perceptions of negative experiences of healthcare by patients with little or no English: a qualitative study across seven language groups	- Poor language skills	49 immigrant patients of a tertiary hospital	Physical and mental health	Australia
19	2012	Correlates of Depression among Chinese Immigrant Elders in Arizona: The Role of Acculturative Stress and Social Support	- English language proficiency	120 Chinese immigrants Elders in Arizona	Mental health	USA
20	2017	The Impact of Being a Migrant from a Non-English-Speaking Country on Healthcare Outcomes in Frail Older Inpatients: an Australian Study	- Culturally and Linguistically Diverse	2,180 immigrant patients of a Liverpool Hospital in south-western Sydney	Physical and mental health	Australia
21	2021	Barriers to accessing preventive health care among African-born individuals in King County, Washington: A qualitative study involving key informants.	- Language - Cultural differences between patients and providers - Lack of cultural emphasis on prevention	16 African-born individuals in King County, Washington	Physical and mental health	USA
22	2022	Barriers and facilitators to cervical cancer screening for women from culturally and linguistically diverse backgrounds; a qualitative study of GPs.	- Patients’ cultural understanding regarding healthcare and cervical cancer screening (CCS) - Communication and language	12 general practitioners’ with experience in providing CCS to women from culturally and linguistically diverse backgrounds	Physical health	Australia
23	2022	Cultural attributes of suicidal ideation among older immigrants: a qualitative study	- Linguistic and cultural barriers of being integrated to the receiving communities - Acculturation gaps in intergenerational support	57 Older Chinese immigrants in Chicago	Mental health	USA
24	2022	Enhancing patient participation of older migrant cancer patients: needs, barriers, and eHealth	- Low health literacy and language barrier	19 Turkish-Dutch and Moroccan-Dutch cancer patients and 12 professionals	Physical health (Cancer treatment)	The Netherlands
25	1995	Social and cultural determinants of smoking behavior in selected immigrant groups: Results of key informant interviews	- Social activity	10 immigrants in the Ottawa-Carleton region from African, Somalian, Lebanese, Iranian, Chinese, Cambodian, Vietnamese, Eastern European, Polish	Physical health.	Canada
26	2004	The effect of acculturation and social support on change in mental health among young immigrants	- Ethnic culture competence - Host culture competence - Ethnic identity crisis - Experiences of discrimination	137 Immigrant students (children with both parents born outside Norway)	Mental health	Norway
27	2012	Conduct Problems and Depression among Unaccompanied Refugees: The Association with Pre-Migration Trauma and Acculturation	- Culture competence	566 Unaccompanied refugees who had been granted residence in Norway	Mental health (psychological well-being)	Norway
28	2021	Trauma and cultural values in the health of recently immigrated families	- Hispanic cultural values	97 immigrants from Guatemala, Honduras, El Salvador, and Mexico	Physical and Mental health (Hispanic health resilience by assessing trauma (exposure and symptoms) in relation to the physical health)	USA
29	2022	Time in the United States and diabetes among Mexican immigrant women: The moderating role of culture	- Level of cultural consonance	18 Mexican immigrant women in Birmingham, Alabama	Physical health	USA
30	2014	Chinese American parents’ acculturation and enculturation, bicultural management difficulty, depressive symptoms, and parenting	- Acculturation and enculturation - Bicultural management difficulty	1,377 Chinese American parents	Mental health (Depressive symptoms)	USA
31	2017	Cultural adaptation, parenting and child mental health among English speaking Asian American immigrant families	- Biculturalism of parents, - Parenting and child development that included cultural adaptation, - Parenting and child outcomes, - Parents’ cultural adaption	157 Asian American immigrant families of children enrolled in early childhood education programs in low-income, urban neighborhoods	Mental health	USA
32	2004	Cultural issues in disease management for Chinese Americans with type 2 diabetes	-The conceptualization of diabetes, illness and health	32 Chinese American patients and spouse	Physical health (Conceptualization of diabetes, illness and health)	USA
33	2011	Cultural beliefs and coping strategies related to childhood cancer: The perceptions of South Asian immigrant parents in Canada	- The cultural beliefs of parents	25 South Asian primary parents	Physical health (Pain of childhood cancer patients)	Canada
34	2009	Cultural predictors of physical and mental health status among Mexican American women: A mediation model	- Ethnic peer affiliation	561 Mexican descent women	Physical and mental health (psychological and physical well-being)	USA
35	2022	‘Cost, culture and circumstances’: Barriers and enablers of health behaviors in South Asian immigrants of Australia.	- Racist bullying	29 Adult immigrants (aged 18 or above) South Asian immigrants	Physical health (physical activity, healthy eating)	Australia
36	1986	Immigrant suicide in Canada: 1971 and 1981	- Social integration	4,000,000 American immigrant cases	Mental health (suicide)	Canada
37	2013	Factors associated with intensiveness of use of child preventive health services in Taiwan: a comparative study between cross-cultural immigrant families and native-born families	- Cross-cultural families	318 Immigrant mothers and 340native-born mothers of children aged 7 years or younger in a cross-sectional survey in central Taiwan	Physical health	Taiwan
38	2017	Health Care Satisfaction: Effects of Immigration, Acculturation, Language	- Ethnicity - Assimilation	383 English-, Spanish-, and Portuguese-speaking immigrants	Physical and mental health	USA
39	2021	Identity-related factors affecting the mental health of African immigrant youth living in Canada	- Presence or absence of a support system - Discrimination - Othering(bullying) - Their personal struggles with Their identity	8 African Immigrant youth in Canada	Mental health	Canada
40	1996	Refugees’ and immigrants’ mental health: Association of demographic and post-immigration factors	- Post Immigration factors (Discrimination, Not having close friends, Being unemployed, Spending most of time with one’s own ethnic groups)	129 Southeast Asian refugees, 57 Pacific Island immigrants, 63 British immigrants	Mental health (anxiety and depression)	New Zealand
41	2010	Perception of barriers to immunization among parents of Hmong origin in California	- Socioeconomic position	417 parents of Hmong origin	Physical health (prevention services such as immunization)	USA
42	2011	Dimensions of acculturation: Associations with health risk behaviors among college students from immigrant families	- American orientation : American practices, Horizontal individualism, Vertical individualism, Independence, American identity - Heritage orientation : Heritage practices, Horizontal collectivism, Vertical collectivism, Interdependence, Heritage identity	3,251 first- and second-generation immigrants undergraduate students	Physical and mental health	USA

## 3 Results

**Figure 2:**
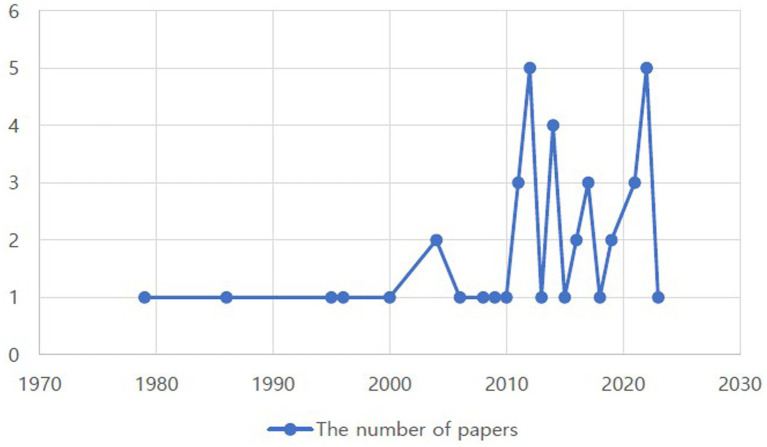
Distributions of papers regarding cultural factors influencing immigrants’ health.

**Figure 3:**
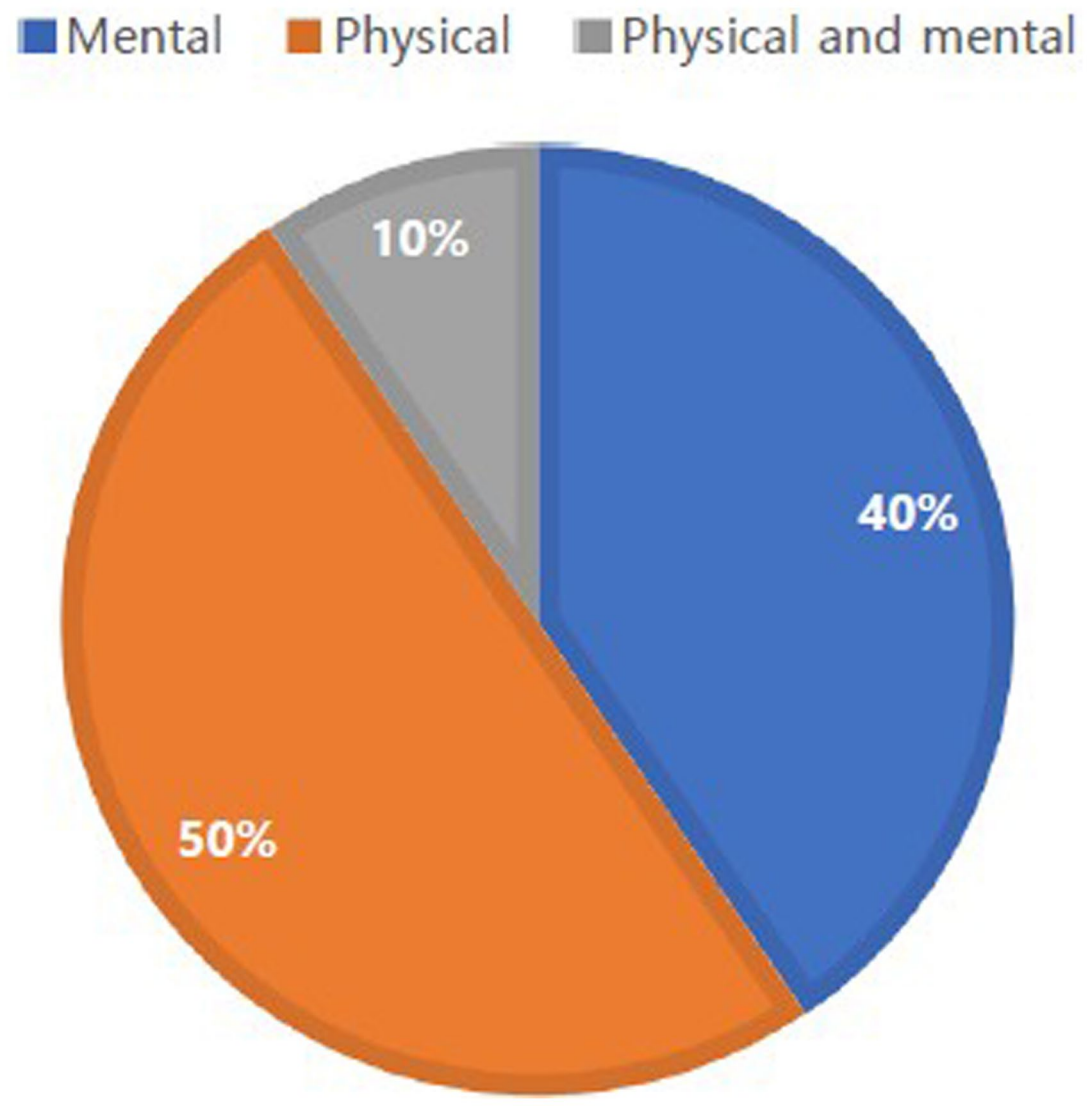
The number of papers investigating physical and mental health of immigrants.

Through a scoping review, 42 studies were examined to identify cultural factors influencing immigrants’ health and were categorized using the Donabedian SPO model (9). The literature for this analysis is represented by year. Between 2010 and 2023, many studies emphasized cultural factors influencing the health of immigrants (Figure 2). The numbers of studies examining physical and mental health are shown in Figure 3. Of the 42 studies, 15 (50%) focused on physical health, 15 (40%) focused on mental health, and 12 (10%) focused on both. No studies have addressed the cultural factors that influence immigrants’ social health. Previous studies were conducted primarily in the United States of America [*n* = 25 (57.8%)], Canada [*n* = 5 (11.9%)], Australia [*n* = 4 (9.5%)], Norway [*n* = 2 (4.8%)], New Zealand [*n* = 1 (2.4%)], Taiwan [*n* = 1 (2.4%)], Thailand [*n* = 1 (2.4%)], Israel [*n* = 1 (2.4%)], the Netherlands [*n* = 1 (2.4%)], and South Korea [*n* = 1 (2.4%)].

### 3.1 Structural factors

Structural factors consisted of social (support systems and social discrimination) and personal factors (ethnicity, language barriers, cultural competence, and parents’ culturalism) that affected the inherent conditions of immigrants.

#### 3.1.1 Support system

Social support is considered a significant factor in promoting immigrants’ mental health and participation in preventive health behaviors, especially for those affected by a lack of appropriate support (15) in their host society. According to Lubben (16), social support is an emotional and instrumental aid derived from interpersonal relationships. Familial and informal social networks of immigrants are often critical for obtaining the information, resources, and emotional support required to adapt to new environments. Social network support is associated with reduced levels of depressive symptoms in older Korean immigrants (17) and fewer mental health issues (18) among African immigrant youths (19). As an example of a social network, Trovato and Jarvis (20) demonstrated that immigrants with religious backgrounds have high levels of social integration, mitigating acculturative stress.

#### 3.1.2 Social discrimination

Social discrimination affects the physical activity and mental health of immigrants. Discrimination has been described in various forms, ranging from microaggression to bullying and ignorance (19). Racist bullying–mostly menacing, insulting, and offending–has been identified as a stressful barrier to physical activity among South Asian immigrants in Australia (21). The experience of discrimination affects anxiety, depression (22), distress, and self-esteem among immigrants (23).

#### 3.1.3 Ethnicity

Ethnicity influences mental health and risky health behaviors. Immigrant patients who rated their ethnicity or culture as important in the context of healthcare decision-making were significantly satisfied with the health guidance they received (24). Hispanic cultural values moderate trauma-health relationships exclusively for adults (25). Mexican American females with high levels of ethnic pride reported elevated levels of family support, which predicted lower levels of mental health issues (26). The authors highlighted ethnic pride as another important cultural factor that should be considered in research on ethnic minority populations (27). For instance, heritage practices and collectivist values are generally protective against health-risk behaviors (27). Chinese immigrants who maintain cultural beliefs about luck and fate were more likely to consume herbal remedies (28).

#### 3.1.4 Language barriers

Language barriers are common challenges for effective healthcare, as reported by immigrants and healthcare providers. In a study investigating the negative experiences of healthcare in Sydney, Australia, immigrant patients with little or no understanding of English reported an inability to communicate effectively (29). Low levels of English proficiency are significantly associated with an increased risk of depression among older Chinese immigrant older adults in Arizona (30) and are more likely to die in hospital (31).

#### 3.1.5 Cultural competence

Cultural competence affects immigrant health. Ethnic cultural competence is assumed to increase adaptation within one’s ethnic and cultural dimension (23). Additionally, cultural competence includes complex cognitive, affective, and behavioral processes, obtaining information about languages, motivational and attitudinal issues, and skills to utilize the acquired knowledge in an appropriate way and situation (32). Oppedal et al. (23) highlighted that ethnic and host competencies promote socio-cultural integration and mental health. Similarly, the construct of cultural consonance is defined as the “degree to which individuals approximate, in their own beliefs and behaviors, the prototypes of beliefs and behaviors encoded in cultural models” (33). Mexican immigrant females in Birmingham, Alabama, with low consonance are likely to be affected by diabetes (34).

#### 3.1.6 Parents’ culturalism

Parenting cultural values and behaviors are related to various aspects of children’s mental health. Banerjee et al. (35) described South Asian immigrant parents’ cultural beliefs about childhood cancer as incurable, rare, unspeakable, and understood through religion, including practicing religious rituals and prayers, indicating the importance of healthcare providers in understanding immigrant parents’ culturalism. Parents’ cultural value of independence appears to be especially salient and negatively related to the behavioral problems associated with immigrant children (36). Baker et al. (37) reported that parents of Hmong origin in California perceived immunization as unimportant for their children, resulting in immunization inequality.

### 3.2 Process factors

The process factor was acculturation, which embraces emotional, behavioral, and affective aspects.

#### 3.2.1 Acculturation

Acculturation has been described positively as a developmental process toward gaining competence within multiple sociocultural settings (23). Acculturation is also the degree to which an individual acquires new culture in terms of cultural behaviors, beliefs, and values (38). Acculturation is positively associated with the identification of mental disorders and negatively associated with personal stigma across generations of immigrants (39). Consedine et al. (40) focused on emotional acculturation because adapting to new cultural contexts requires massive and complete reconfiguration and recalibration of basic systems. In their study, female immigrants in the USA, who were emotionally different (less acculturated), reported significant somatic symptoms. Behavioral and affective acculturation are indirectly associated with depressive symptoms through perceived discrimination (41).

### 3.3 Outcome factors

Outcome factors included acculturative stress and ethnic identity crisis.

#### 3.3.1 Acculturative stress

Acculturative stress is the physiological and psychological state of an individual caused by culture-specific stressors rooted in the acculturation process (42). Elevated levels of acculturative stress are significantly associated with high levels of C-reactive protein and soluble tumor necrosis factor receptor 2 in Chinese immigrant females (43). Mui and Kang (44) investigated older American and Asian populations in the US and Asia and reported that acculturative stress caused by older people’s perception of a cultural gap between themselves and their adult children was associated with high levels of depression. A study on Asian Americans in the US reported that acculturative stress was a significant predictor of mental health (45).

#### 3.3.2 Ethnic identity crisis

Ethnic identity crises have been largely assessed among immigrant youth. During acculturation, individuals go through the process of developing their migrant youth identity. A significant ethnic identity crisis has been correlated with great distress and low self-esteem among immigrant youth in Norway (23). In Canada, the mental health of American immigrant youth is affected by their struggles with their identity (19).

## 4 Discussion

This study analyzed the cultural factors related to immigrants’ integrated health, encompassing physical, mental, and social aspects. Cultural factors were categorized and presented using Donabedian’s SPO model to provide a structured understanding of these factors.

First, social (social support and discrimination) and personal (ethnicity, language barriers, cultural competence, and parenting) factors were identified as structural factors. This finding is consistent with those of previous studies (46). Structural and social factors play important roles in immigrants’ health outcomes (17–19). Individual health resources such as health knowledge and self-efficacy have been associated with improved health outcomes in newly resettled refugee migrants, emphasizing the importance of support systems that buffer structural barriers to health (46). These similarities can be attributed to enhancing social support and addressing social discrimination to improve immigrants’ health and well-being. Social support can be obtained from inside (17) and outside of family (19). Religious involvement has been introduced as the largest source of social support (19). Therefore, in order to improve immigrants’ health outcomes, developing various religious activity program based on their religious needs should be considered as a strategy.

Other structural factors include ethnicity, language barriers, cultural competence and parental culture. Cultural competence and parental cultural orientation have been identified as personal factors among immigrants. Previous studies highlighted the importance of cultural competence as an essential skill for medical professionals, with training programs suggested to enhance this competency for physicians, nurses, and mental health professionals (47, 48). Relatively few studies have analyzed cultural competence in immigrant health. However, considering that immigrants with low levels of cultural competence are likely to suffer from diabetes (32), cultural competence has emerged as a crucial factor affecting their health. This highlights the need for future studies to explore strategies for enhancing cultural competence.

Acculturation is a key factor in this process. Research on the cultural adaptation of immigrants demonstrates that acculturation and enculturation are multidimensional and context-dependent cultural socialization processes, highlighting the intricate nature of cultural adaptation (49). Acculturation is regarded as a process factor in studies that does not specifically address immigrant health. Recent studies have demonstrated that acculturation can have ambivalent effects on the mental, physical, and cognitive health of immigrant populations (50, 51). Additionally, acculturation is closely linked to the experience of racism, which is a significant social determinant of immigrant health (52). That is, addressing acculturation pathways is important to mitigate the impact of racism on immigrants’ health. Acculturation manifests initially through behavioral changes (e.g., language use, diet), followed by changes in values or identity (49, 53). This sequential pattern suggests that lifestyle changes such as dietary life that occur in the early acculturation phase may have important implications for immigrant health. Thus, early intervention in the behavioral adaptation phase may have important policy implications for advancing health equity and shaping preventive health policies.

The outcome factors were acculturative stress and ethnic identity crisis. The negative effects of acculturation and acculturative stress, when not managed effectively, can result in adverse health consequences such as hypertension and obesity, particularly affecting Black and Hispanic immigrants (54). Mui and Kang (44) revealed that acculturative stress causes generational gaps between parents and children, indicating that stressors can originate from both intercultural and intrafamilial differences. Psychological strain is associated with adverse mental health outcomes and stress-related physical conditions, highlighting the need for culturally sensitive support systems and interventions (54, 55). Culturally tailored services such as family-based acculturation intervention have been reported to reduce acculturation stress and improve immigrants’ health outcomes (56). These findings support the need for policies that ensure access to culturally competent, family-focused health care for immigrants populations.

Although this study attempted to analyze the cultural factors that affect overall health, many studies have focused on physical and mental health. However, social health issues have not been addressed yet. Social health is defined as the adequate quantity and quality of relationships within a specific context to fulfill an individual’s need for meaningful human connections (57). Furthermore, social health plays a significant role in addressing health inequalities among marginalized groups and is essential for understanding population health concerns (57, 58). Given the pivotal role of social health, future research should prioritize the underexplored areas of immigrants’ social health. Addressing social health as an integral component of immigrant well-being can lead to more inclusive and equitable public health systems.

## 5 Limitations

This study was limited by the disproportionate number of studies written in English (41 out of 42), with only one study published in Korean. As a result, the findings may be less generalizable to non- English-speaking contexts. Cultural factors that affect immigrants’ health may differ in the early, middle, and late stages of settlement, or by era; however, this study could not distinguish between these gaps. Further studies are required to identify these gaps. Additionally, one notable limitation of this review lies in the search strategy, which primarily relied on terms such as “health,” “healthcare,” and “medical.” As a result, studies that conceptualize social health using alternative terms such as “well-being,” “social connection,” or “loneliness” may have been unintentionally excluded. Future reviews could consider including the keywords to more comprehensively capture the multidimensional nature of health, including its social components. Lastly, this study focused on cultural factors and did not include socioeconomic conditions, which may also influence immigrant health but fall outside the scope of our conceptual framework. Future research may benefit from exploring how cultural and socioeconomic factors interact to shape health outcomes in immigrant populations.

## 6 Conclusion

This scoping review underscores the critical role of cultural factors in shaping the health outcomes of immigrant populations. By applying Donabedian’s SPO model, this study revealed structural factors, such as social support, social discrimination, ethnicity, language barriers, cultural competence, and parenting, all of which significantly influence immigrant health. These findings are consistent with previous studies and highlight the critical role of structural factors in mitigating immigrants’ health problems. This study emphasizes the importance of fostering social support systems and addressing social discrimination to enhance immigrant populations’ overall well-being, thereby informing the development of culturally responsive policies and providing a robust evaluative framework grounded in the SPO model.

Additionally, this study identified acculturation as a crucial process factor and explored its complex relationship with health risks and racism. This study highlights the adverse health effects of acculturative stress and ethnic identity crises, and emphasizes the need for culturally sensitive support systems. Despite the extensive focus on physical and mental health, this study revealed a notable gap in the research on immigrants’ social health. Given the pivotal role of social health in mitigating health inequalities and enhancing the quality of life, future research should prioritize this area to develop comprehensive strategies that address the holistic health needs of immigrants. By expanding the scope of the research to include social health, policymakers and healthcare providers can reduce health disparities and promote equity of immigrant communities.
